# Preclinical coronavirus studies and pathology: Challenges of the high-containment laboratory

**DOI:** 10.1177/03009858221087634

**Published:** 2022-04-11

**Authors:** Victoria K. Baxter, Stephanie A. Montgomery

**Affiliations:** 1 The University of North Carolina at Chapel Hill, Chapel Hill, NC

**Keywords:** coronavirus, SARS-CoV-2, preclinical model, specimen handling, biosafety, high-containment, BSL3

## Abstract

The COVID-19 pandemic has highlighted the critical role that animal models play in elucidating the pathogenesis of emerging diseases and rapidly analyzing potential medical countermeasures. Relevant pathologic outcomes are paramount in evaluating preclinical models and therapeutic outcomes and require careful advance planning. While there are numerous guidelines for attaining high-quality pathology specimens in routine animal studies, preclinical studies using coronaviruses are often conducted under biosafety level-3 (BSL3) conditions, which pose unique challenges and technical limitations. In such settings, rather than foregoing pathologic outcomes because of the inherent constraints of high-containment laboratory protocols, modifications can be made to conventional best practices of specimen collection. Particularly for those unfamiliar with working in a high-containment laboratory, the authors describe the logistics of conducting such work, focusing on animal experiments in BSL3 conditions. To promote scientific rigor and reproducibility and maximize the value of animal use, the authors provide specific points to be considered before, during, and following a high-containment animal study. The authors provide procedural modifications for attaining good quality pathologic assessment of the mouse lung, central nervous system, and blood specimens under high-containment conditions while being conscientious to maximize animal use for other concurrent assays.

As novel pathogens emerge and new animal models requiring high-containment facilities are developed, these principles can guide pathologic assessment of future models. Not only was the scientific community able to rapidly pivot in response to the COVID-19 pandemic, but it was also a defining moment for animal models, which proved to be an invaluable tool for rapid evaluation of therapeutics, from remdesivir to monoclonal antibody treatment. By their very nature, pathology data sets generated from animal models are translational research and crucial measures for evaluation of preclinical models. Over recent years, the pathology community has worked to establish technical guidance promoting best practices to support pathologic end points and promote rigor and reproducibility,^30,31^ but these very practices pose challenges when operating within the constraints of a high-biocontainment laboratory, where coronavirus studies are typically conducted. The aim of this article is to highlight challenges in conducting coronavirus preclinical studies and consider work-around approaches to incorporate pathological end points. As with all studies, advanced planning and communication among investigators and pathologists, followed up with transparency in reporting results, will promote the best science and use of animals.

## High-Containment Laboratories

Different biohazardous agents are assigned a biosafety level (BSL) at which work with these agents may take place following an assessment of biological safety risk.^8,37^ In the United States, there are 4 BSLs, designated 1–4, with biosafety facilities, safety equipment, practices and procedures, and personal protective equipment (PPE) increasing in stringency as the BSL increases.^8^ Biohazardous agents designated for work at BSL1 do not typically cause disease in immunocompetent individuals and include agents such as *Bacillus subtilis*, *Saccharomyces cerevisiae*, and laboratory strains of *Escherichia coli*. Work with these agents may be conducted using basic safety precautions and standard microbiological practices in laboratories that do not contain specialized primary or secondary barriers outside of a door, a handwashing sink, and easily cleanable work surfaces. BSL2-designated agents have the potential to cause disease in immunocompetent humans with varying degrees of severity primarily through percutaneous, mucosal, or oral routes of exposure and include pathogens such as seasonal influenza A viruses, *Staphylococcus aureus*, and *Toxoplasma gondii*. In addition to the standard precautions taken at BSL1, and in line with the risks associated with working with BSL2 agents, extra precautions are taken when working with sharps, and procedures with high potential for splashes or aerosolization are generally conducted using biosafety cabinets (BSCs) or other physical containment equipment.

The term “high containment” is reserved for BSL3 and BSL4 practices and facilities.^13^ Examples of agents designated for work at BSL3 include *Mycobacterium tuberculosis*, SARS-CoV-2, and yellow fever virus, which have the potential to cause severe or lethal disease following aerosol transmission.^8^ Compared with BSL1 and BSL2, more emphasis is placed on primary and secondary barriers at BSL3 to protect the personnel, the surrounding community, and the environment. To minimize the risks associated with aerosol transmission, workers must wear respiratory protection such as N95 masks or powered air-purifying respirators (PAPRs), and all work must be conducted in a BSC or using a primary containment device, such as a centrifuge with rotor gasket and sealable cups. BSL3 facilities must also employ advanced ventilation systems with negative directional airflow and measures to limit access.

BSL4 agents are considered the most dangerous pathogens, with the high potential of causing life-threatening disease typically for which no vaccine or effective therapeutic is available, or if a breach in biocontainment would create a severe risk to the community given that most of the population is unvaccinated. BSL4 pathogens include filoviruses, such as Ebola virus and Marburg virus, and the henipaviruses. Working safely with BSL4 agents requires individuals to use highly specialized practices and safety equipment, such as 100% contained Class III BSCs or full-body, air-supplied positive-pressure suits. BSL4 facilities are often separate buildings with specialized ventilation and waste management facilities, and for these reasons, only 12 operational or planned facilities exist in the United States.^17^ BSL4 laboratories operate with several layers of safety redundancy to ensure functionality of critical safety engineering systems, such as air handling and effluent decontamination, which is colloquially known as “N + 2 engineering,” indicating 2 backup systems for each primary unit.

The large jump in facility design parameters and safety precautions and practices needed when working with BSL3 and BSL4 agents requires special considerations and arrangements so that experiments may be conducted safely. Guidance on the design and operation of high-containment facilities is primarily provided by the *Biosafety in Microbiological and Biomedical Laboratories* in the United States^8^ and the *Laboratory Biosafety Manual* published by the World Health Organization at the international level.^37^

While this article focuses on performing work in BSL3 facilities, it is important to note that BSL4 facilities provide an alternative resource for conducting SARS-CoV-2 studies. BSL4 laboratories have large footprints, are generally better funded than most BSL3 labs through support of operations grants and reimbursement through large governmental and industry contracts, have established standard operating protocols (SOPs) that are immediately translatable to lower pathogens that are not Select Agents such as SARS-CoV-2, and have access to advanced technologies and a wealth of highly skilled personnel, including animal care staff, that can conduct all levels of translational research.

Compared with BSL1 and BSL2 facilities, regulatory oversight is more involved for personnel working in BSL3 facilities both within and outside the institution.^11,13^ Within the institution, biosafety practices for work in the BSL3 facility can be overseen by several departments or committees, such as Environment Health and Safety or Occupational Health and Safety for general laboratory oversight and personnel training, the Institutional Biosafety Committee (IBC) for work involving recombinant nucleic acids, and the Institutional Animal Care and Use Committee for work involving animals.^11^ Any institution that receives funds from the National Institutes of Health (NIH) and performs work with recombinant nucleic acids at BSL3 or BSL4 is required to employ a biological safety officer, to oversee lab inspections, advise on biosafety and biosecurity, and report any problems.^22^ No one federal entity oversees high-containment laboratories in the United States, and depending on the nature of the work being performed, high-containment laboratories are subject to regulations outlined by a variety of agencies, such as the Centers for Disease Control and Prevention (CDC), Department of Defense, NIH, US Department of Agriculture (USDA), Environmental Protection Agency, Federal Drug Administration, National Institute for Occupational Safety and Health, Department of Transportation, Federal Aviation Administration, and Occupational Safety and Health Administration, in addition to state and local departments of health.^1,28^ Animal work at BSL3 and BSL4 is overseen by several organizations including USDA Animal and Plant Health Inspection Service, Office of Laboratory Animal Welfare, and AAALAC International.Pathogens deemed to pose an especially high risk to human, animal, or plant health with concern for weaponization are classified as Select Agents.^5^ This designation automatically applies to all BSL4 pathogens but only to some of the BSL3 pathogens. Examples of BSL3 Select Agents include bacteria such as *Yersinia pestis*, *Francisella tularensis*, *Bacillus anthracis*, and *Brucella* spp., and viruses such as Rift Valley fever virus, eastern equine encephalitis virus, Venezuelan equine encephalitis virus, reconstructed 1918 influenza virus, and SARS-CoV. Work with Select Agents requires registration with the CDC and/or the USDA as part of the Federal Select Agent Program and involves increased documentation, security procedures, and periodic inspections.^5,10^ These regulations are in place to ensure biosafety and biosecurity, but effort from individuals at multiple institutional levels is required for high-containment facilities to remain in compliance with the appropriate regulatory oversight bodies. Such regulatory oversight comes with increased bureaucratic burden in handling specimens generated in a high-containment laboratory, requiring tracking records and signatures for transferring out of high-containment laboratory and all transfers over the life of the specimen through its disposal, which itself often has special requirements.

**Figure 1.**
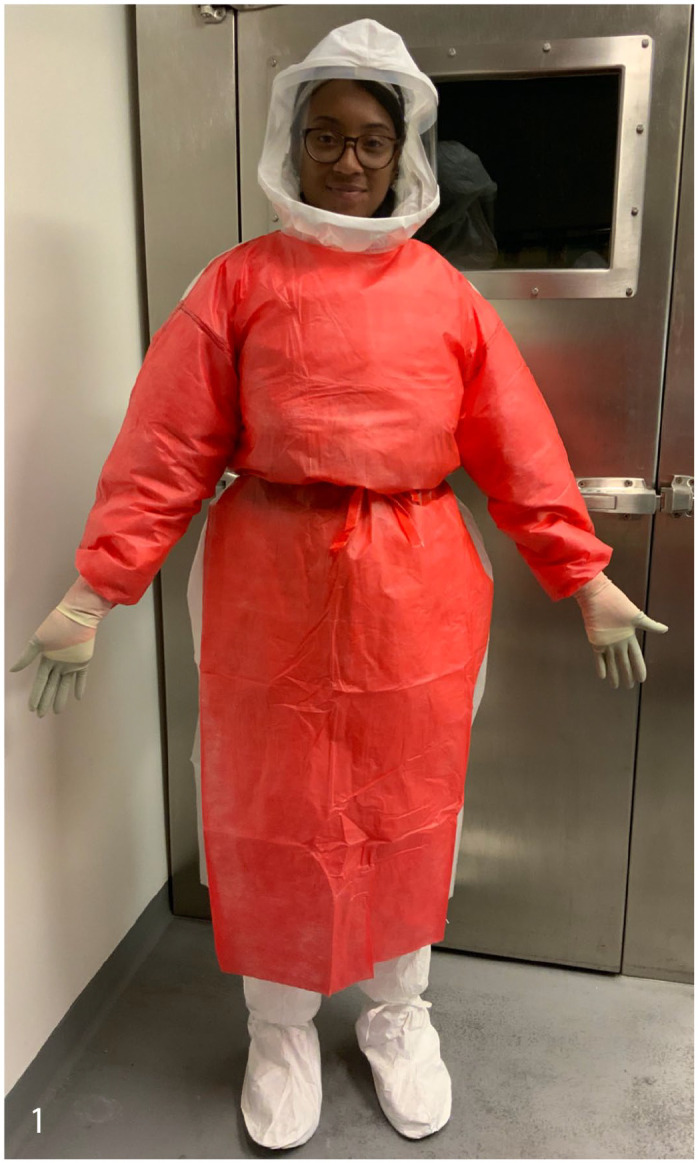
Typical personal protective equipment worn in a BSL3 facility, including powered air-purifying respirator, Tyvek suit, solid-front gown, double gloves, and shoe covers.

**Figure 2.**
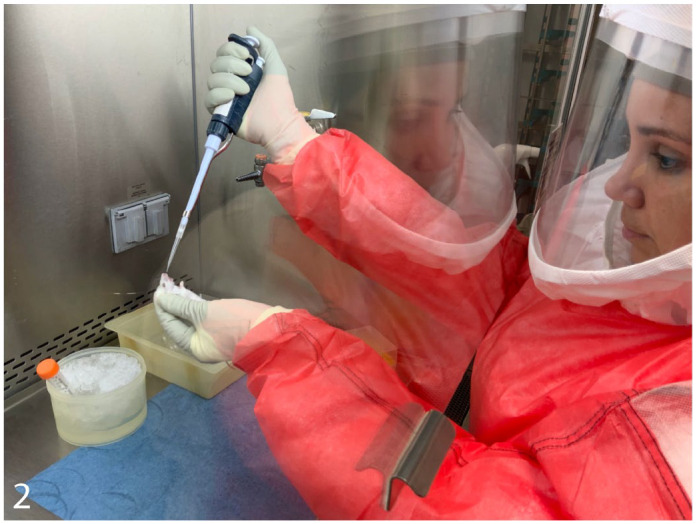
Conducting animal studies in a biosafety cabinet in a BSL3 facility.

Beyond the goals of an experimental study, protection of personnel from accidental pathogen exposure (biosafety) and prevention of pathogen escape (biosecurity) are 2 primary objectives when working with pathogens in the BSL3.^28^ BSL3 facilities are required to have limited access,^8^ and personnel undergo extensive training, estimated to cost up to $7000 per person, and must demonstrate proficiency in working with the BSL3 agent before they are allowed to operate in the facility by themselves.^1^ Therefore, only a few select personnel are qualified at any one time and able to conduct an animal study in the BSL3. Working in the BSL3 can be tiring, and personnel must balance working the necessary hours to complete a task or experiment with taking appropriate breaks to reduce fatigue-induced mistakes that could result in pathogen exposure. The PPE required at BSL3 is more extensive than at BSL1 or BSL2, including respiratory protection in the form of an N95 mask or PAPR, a solid-front liquid-impermeable gown, and double gloves (Fig. 1).^8,29^ Animal procedures are performed within a BSC where a sash separates the user from the animal, and to not disrupt laminar airflow, work is performed in the center of the BSC surface; this often results in the animal being 30 cm or more away from the user’s body when performing manipulations, which can contribute to user fatigue and eye strain. Coupled with wearing a PAPR, where the user’s face is several centimeters from the PAPR shield, the ability to perform close-up work is limited in the BSL3 (Fig. 2). While PPE offers such safety to the user, it comes with the trade-off of restricting fine movements and imposing communication challenges created by ventilation noise and face shield barriers.

Because aerosolization of infectious agents is a major risk when working with BSL3 agents, many of the mitigation efforts center around reducing aerosolization. Aerosol-producing activities, including rapidly removing gloves, vigorously mixing reagents, blowing out pipettes or needles, and using vacuum and aspiration equipment, are minimized.^37^ Risks associated with aerosol-producing activities that cannot be avoided, such as changing animal cages, infecting or treating animals via intranasal inoculation, and performing necropsies of infected animals, are mitigated through specific handling techniques and the use of BSCs and other primary containment devices.^8,14,23,33^

BSL3 facility layout and space also affect the way in which animal experiments are conducted. BSL3 labs are usually small, and depending on the layout of the facility, animal housing and procedure space is often in the same room. If multiple rooms are present in a facility, they tend to be modular and small to allow separation of workspaces and optimize ventilation; this limits the number of animals that can be housed in the space, particularly nonrodent species. Furthermore, depending on the nature of the study, animals may need to be singly housed due to transmission concerns, further restricting animal housing numbers. Therefore, with space for animal housing often at a premium, maximizing informational output from a single animal is beneficial when planning BSL3 animal experiments.

**Figures 3–6.**
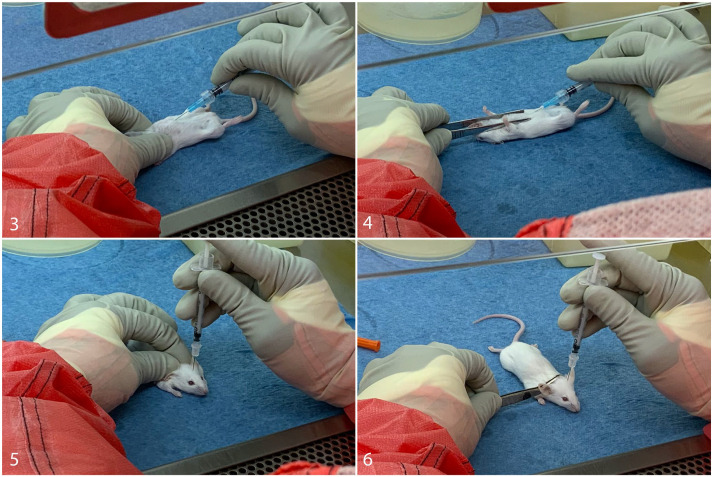
Examples of standard hands-on versus preferred hands-free techniques when using sharps with animals in a BSL3 facility. **Figure 3.** Cardiocentesis was performed with the nondominant hand stabilizing the mouse body. **Figure 4**. Cardiocentesis was performed with forceps stabilizing the mouse body. **Figure 5.** Intracranial inoculation was performed with the nondominant hand stabilizing the mouse head. **Figure 6**. Intracranial inoculation was performed with forceps stabilizing the mouse head.

Conducting animal experiments in BSL3 facilities involves several logistical challenges that can impact the collection of tissues and samples and subsequent pathologic analysis. Animal infections, monitoring, and tissue collection usually take longer at BSL3 compared with at BSL1 and BSL2, and therefore fewer animals can be processed in a single day. The use of sharps, including glass pipettes, needles, scalpels, razor blades, and bone-cutting electric saws, is strongly discouraged in the BSL3 lab due to the risk of pathogen exposure posed by an accidental cut or needle stick.^8^ When using needles is unavoidable, such as with intracranial injections or cardiocentesis, modifications to the technique may be employed, such as using forceps to stabilize the animal’s head or body instead of using one’s fingers (Figs. 3–6), using needle blocks, and placing sharps containers directly within the line of sight for immediate disposal. While these modifications minimize the risk of needle stick, the stabilization of the body or head is typically not as secure when using forceps during needle insertion, commonly introducing some tissue handling artifacts. Furthermore, injectable anesthetics are not considered a first choice in sedation, and the increased use of inhalant anesthetics such as isoflurane can affect study outcomes, particularly when the respiratory or neurological systems are being evaluated.^16,34,35^

The use of tools such as bite-resistant gloves or rubber-tipped forceps is encouraged when handling animals to minimize the risk of pathogen exposure through bites;^4^ however, these tools, coupled with the double surgical gloves, or cut-resistant gloves between double-layered surgical gloves, further reduce user dexterity. When working with fractious animals with a heightened bite or scratch risk, animals are often sedated to facilitate safer handling. Depending on the frequency at which animals need to be handled, animals may undergo multiple sedation events throughout a study, and this in turn may affect experimental results and tissue samples.^16^ Investigators try to control for these effects by treating all animals in the study in the same manner.

Because all work must be performed in a BSC or using some other form of primary containment,^8^ availability of the BSC is often the limiting factor when conducting BSL3 studies. In most cases, all animal procedures, monitoring, euthanasia, and tissue collection take place within the BSC. If multiple pathogens are used in the same BSL3 space, thorough decontamination of BSC space is required between working with animals infected with the different pathogens,^8^ which slows the process. Furthermore, smaller equipment such as homogenizers, ELISA analyzers, and hematology analyzers may not be permanently housed in BSCs, requiring personnel to physically move the equipment. Larger or heavier equipment may require a different primary containment system, such as a soft-wall containment enclosure. These enclosures often have a large footprint, further taking up floor or bench space already at a premium. Therefore, the variety and availability of equipment is often limited in the BSL3 lab and tailored toward the experiments commonly performed in the facility. Any equipment housed in the BSL3 facility must be thoroughly decontaminated with freshly prepared 10% bleach, or a specific IBC-approved chemical, such as a hospital-grade detergent-disinfectant formulated cleaner, at the appropriate contact time before it can be removed from the lab.^8^ Therefore, readily moving equipment back and forth between the standard lab and BSL3 lab for single experiments is not regularly done. Certain pieces of equipment require calibration with non–shelf-stable reagents immediately prior to running samples, such as a hematology analyzer, or use large reagent plates for multiple samples, such as many multiplexing assay analyzers; it is not financially feasible to run one or two samples on these pieces of equipment, and therefore, individual samples are often stored until a large enough number are obtained, which can affect assay outcome.^32^ Storing items in containers that cannot be easily decontaminated such as Styrofoam or cardboard is also discouraged, and so personnel minimize the number of reagents and supplies kept in the BSL3 and instead bring in only the items they need for the experiment on which they are actively working. Therefore, animal experiments and collection of tissues for pathology in the BSL3 lab require advanced planning to ensure BSC’s in which to work and appropriate materials and reagents are available and accessible. Making on-the-spot changes to collection protocols is not common practice.

Due to limitations in available bench space and the avoidance of sharps, equipment for embedding, sectioning, and staining tissue specimens is rarely kept in the BSL3 facility, necessitating fixed tissues to be brought out of the lab for further processing. However, for samples to be brought out of the BSL3 lab, any potential infectious agent must first be inactivated.^8^ SARS-CoV-2 can be inactivated in different tissue and fluid samples using several methods, including ultraviolet irradiation, formalin fixation, and heat inactivation.^18,19,24,36^ Federal regulations require that institutions have internally validated inactivation data for all work with Select Agents,^7^ where each proposed inactivation method must be validated in each specific tissue or fluid specimen that will potentially be removed from the BSL3 facility and approved by an internal regulatory group, usually the IBC.^25^ Published and well-established protocols for inactivating nonselect BSL3 agents, such as SARS-CoV-2 and *M. tuberculosis*, can be used to remove samples from the BSL3 facility, but institutions may still require their high-containment laboratories to perform internal inactivation validation studies for certain tissues or when using specific pathogen strains. This process can be time consuming and take several months to complete.

Most validation assays for virus inactivation involve applying homogenized, inactivated sample to cells and monitoring the cells for evidence of plaque formation or cytopathic effects (CPE).^24,36^ For many inactivation solutions, including formalin, detergents, and nucleic acid extraction reagents, residual fixative or extraction reagent in the samples will often induce CPE if the concentration is too high.^2,36^ In these cases, homogenized samples must be diluted to where CPE is no longer induced by the cytotoxic reagent alone, and the institutional regulatory group that reviews inactivation protocols must determine an acceptable assay limit of detection at which they are satisfied any infectious virus has been inactivated. As an alternative approach, the cytotoxic reagent may be removed from the sample using filtration matrices, but this may affect the sensitivity of the inactivation validation assay through pathogen loss or reduction of infectivity.^36^ The lengthy formalin fixation or other inactivation protocols required to remove tissue specimens from a high-containment laboratory may negatively impact downstream tissue assays, such as immunolabeling, *in situ* hybridization, or various nucleic acid analyses. Some of these unwanted effects may be circumvented by transferring tissues from 10% neutral buffered formalin (NBF) to 70% ethanol after a complete 7-day formalin fixation to prevent further protein cross-linking.

Confidence in thorough virus inactivation is especially important when inactivated tissue and specimens are submitted to collaborators and core facilities for further processing and analysis, as these specimens will be handled by personnel unfamiliar, untrained, and potentially uncomfortable with the procedures of high-containment laboratories. When transferred to other laboratories or entities, all inactivated samples from studies involving Select Agents are federally mandated to be accompanied by a certificate of inactivation documenting execution of the institutionally approved inactivation SOP.^7^ While this certificate is only mandatory for Select Agents, its use is standard practice for transfer of all BSL3 pathogens.

## Performing Pathology Studies in the High-Containment Laboratory

### Example: Lungs

**Figures 7–8.**
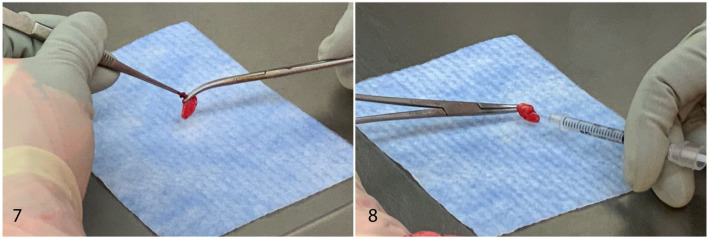
Direct lung lobe inflation, mouse. **Figure 7.** Clamping of the main bronchus with hemostats. **Figure 8.** Instillation of fixative into lung parenchyma with a 29G needle on an insulin syringe.

**Figures 9–10.**
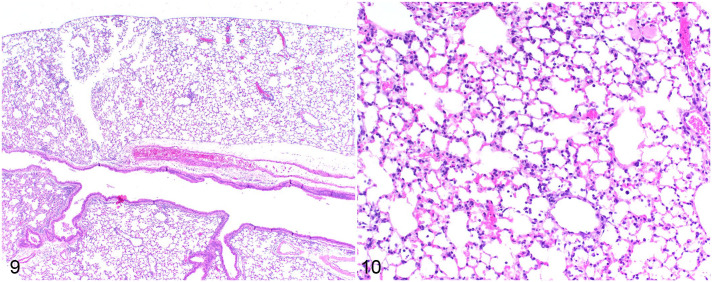
Lung, mouse. Hematoxylin and eosin. **Figure 9.** A needle track artifact is visible. The left lung lobe was directly injected with formalin using an insulin syringe with a 30G needle. **Figure 10.** Typical histomorphology with inflation of alveoli acceptable for pathologic assessment. The lung was inflated by direct injection of air as shown in Figs. 7, 8.

For SARS-CoV-2 infection studies, lungs are commonly collected for histopathological analysis. For reasons previously described, in the BSL3 facility, animals are used to their full potential, with different lung lobes commonly used for different assays, such as virus titers, flow cytometry, and gene and protein expression in addition to histopathology. To concurrently minimize animals dedicated to these studies and maximize information gained from an individual, lungs are removed from the thoracic cavity and each lobe separated for different assays. Among the large coronavirus research group at our institution, lung lobe collection from mice has been standardized, with the left lobe, the largest lung lobe in rodents, reserved for fixation for histopathology. With the lack of air inspiration after death, the delicate alveolar septa tend to collapse on themselves, complicating pathologic assessment. In an effort to preserve lung parenchyma in a physiologically relevant state and aid the pathologist in evaluating disease, particularly of viral interstitial pneumonia, lung insufflation is commonly performed. In a typical laboratory setting, formalin is injected into the trachea to inflate the lungs before removing them from the thoracic cavity for further immersion fixation. The standard inflation method involves injecting formalin rapidly at constant pressure, usually 20–25 cm H_2_O, which opens airspaces without damaging the delicate alveolar sacs.^3,15,27^ If inflating the lungs with formalin using constant pressure is not possible, an alternative option is to inflate the lungs with formalin via the trachea without measuring pressure, though this may result in overinflation.^9^ However, in BSL3 studies where some lung lobes need to be collected to determine the virus titer or other analyses, lung inflation via intratracheal injection is usually not feasible because it results in fixation of all lung lobes. Other lung lobes can be tied off with suture prior to injecting formalin into the trachea, but this method is technically challenging in small rodents such as mice and can result in formalin contamination of other lung lobes. An alternative approach is to use hemostats to clamp the right mainstem bronchus, distally cut the right lung lobes while keeping the hemostat clamped, and then instill formalin into the left lobe. An option for inflating individual lung lobes employed by our BSL3 research group is to separate the lobe of interest from the rest of the lung, clamp the main bronchus with forceps, and slowly inject a small amount of formalin (50–100 mL for the left lung lobe in mice) directly into the parenchyma of the lung lobe using an insulin syringe with a 29G–30G needle (Figs. 7, 8). While this method does result in inflation of the lungs, a needle track artifact is introduced (Fig. 9) and uncontrolled injection pressure and speed can result in artifacts. Another alternative, depending on downstream tissue assays, is to clamp the right bronchus then inject 1% low-melting-temperature agarose and 1X phosphate buffered saline (PBS) into the trachea to inflate the left lung, as agarose and PBS are less likely to negatively impact some analyses such as viral titers in case of contamination. In some cases, inflation of the lung lobe is not an option, resulting in submission of an uninflated sample. However, with gentle handling of the lobe during removal, because of their small size, an inflated rodent lung specimen acceptable for pathologic assessment can be generated (Fig. 10).

### Example: Central Nervous System (CNS) Tissue

It is notoriously difficult to obtain suitable samples of the CNS for histopathology, and neuroscientists and neuropathologists have dedicated much effort into identifying the ideal fixation method for assessing pathology and preparing tissue for special stains and immunohistochemistry.^6^ A common method is intravenous perfusion at physiologic pressure with ice-cold 4% paraformaldehyde (PFA) prior to tissue removal, and brains and spinal cords are then immersed overnight in 4% PFA at 4°C. However, for the appropriate volume of PFA perfused at physiologic pressure (80–120 mm Hg, equating to approximately 2–3 mL/min for mice), the perfusion processing takes roughly 30 minutes per animal, limiting the number of animals that can be processed at one time. Long days in the BSL3 lab can result in investigator fatigue, increasing the risk for accidents, so necropsy efficiency is often prioritized over optimal perfusion techniques. The increased rate of perfusion as well as other manipulations prior to full tissue fixation can result in CNS tissue artifacts of which the pathologist should be aware.^12^ In addition, many scientists consider a short immersion time at 4°C to be optimal for immunohistochemistry, but this can pose challenges when validating inactivation of the infectious agent.^20^ As mentioned earlier, virus inactivation assays, which are required for removing tissue specimens from the BSL3 facility for further processing and analysis, commonly involve assessing eukaryotic cells for CPE following application of inactivated samples, and residual formalin or PFA in samples will induce CPE if present at high enough concentrations.^24,36^ Gaining regulatory approval of an inactivation method is a cumbersome process that can take months or, in extreme cases, years, and validation of inactivation must be performed for each individual tissue type for every different infectious agent. Adding to the challenge, if multiple different pathogens are used in the same BSL3 space, safety precautions and practices, including inactivation protocols, for the entire space must meet the biosafety standards of the most rigorous agent. Our group has encountered this obstacle firsthand, where 7 days of immersion in 10% NBF are required before SARS-CoV-2-infected tissues can be removed from the BSL3 facility. Therefore, the 7-day NBF fixation protocol has had to be applied for CNS tissues infected with other pathogens until approval can be obtained for overnight 4% PFA immersion fixation as inactivation methods based on testing of SARS-CoV-2-spiked CNS samples. In addition, for several SARS-CoV-2 animal experiments, brains have been collected along with other tissues for neuropathologic assessment. However, because the brain is not the primary organ of interest in these studies, perfusion with 4% PFA has not been conducted in most cases, and all tissues have had to be fixed in 10% NBF for 7 days rather than 4% PFA for less than 24 hours (Fig. 5). Despite less-than-ideal tissue preparation methods, subsequent pathological analysis can be successful.

For optimal brain tissue analysis, a standardized method should be established for consistent evaluation of pathological changes.^21^ The routine approach is perfusion at the time of necropsy, extraction of the brain *in toto* after further immersion fixation, followed by removal from the high-containment facility and gross assessment using a brain matrix. Alternatively, the whole head may be fixed without removal of the brain, though it is critical to remove the skull cap with scissors to allow proper formalin fixation of CNS tissues. After fixation, the head must be decalcified, which could impact future ancillary analyses, particularly *in situ* hybridization. Ethylenediaminetetraacetic acid (EDTA) and formic acid solutions take longer to achieve full decalcification but are less damaging to CNS tissues compared with faster decalcifying solutions such as hydrochloric acid.^26^This tissue preparation method is ideal when performing studies to track neuroinvasion and pathogen spread throughout the CNS in sagittal sections. As SARS-CoV-2 replicates in the nasal turbinates but also induces neurological disease, being able to evaluate both nasal and CNS tissue on the same slide is especially relevant to coronavirus pathogenesis studies. Previously established standardized sampling approaches such as the widely used RENI trimming guide (https://reni/item/fraunhofer.de/reni/trimming) are recommended.

### Example: Blood work

Challenges associated with collecting and analyzing tissues from animals in the BSL3 are not limited to anatomic pathology, but also include clinical pathology. Initially, one might imagine running a complete blood count (CBC) on a hematology analyzer to be straightforward, as the equipment does not have a large footprint, is inexpensive, and often has been optimized for small sample volumes collected from rodents. However, fluid samples often need to be analyzed soon after collection and cannot be stored without compromising downstream analyses,^32^ requiring the assays to be performed in the BSL3 facility. The analyzer itself generates aerosols when taking up blood samples,^38^ requiring them to be housed in a BSC during operation, and the waste generated by the instrument must be decontaminated without compromising the future technical capability of the instrument. Analyzers are small but weigh ~12 kg and are bulky for a user wearing PPE to move in and out of the BSC, if dedicated housing in the BSC is not available. In addition, the quality control reagents required for calibrating the instrument are expensive and add time to the analysis process. CBCs can be run on one sample at a time but can take 2–20 minutes, limiting the number of samples that can be run in a day; running a CBC on a single sample is not usually financially feasible. Because of this, investigators must be mindful when incorporating CBCs and similar analyses as part of an experiment, and in cases where not all samples can be processed in a single day, samples should be properly stratified to minimize batch effects. Another challenge is that blood films are not routinely prepared to confirm the accuracy of CBC results, as they require handling of glass sharps and an approved inactivation SOP for removal from the high-containment laboratory. An alternative is a semiautomated or automated blood film analyzer in the BSC, which represents an added expense and an extra instrument within the facility. When analyzing CBCs and related assays, the pathologist and the investigator should discuss the special conditions and circumstances of the BSL3 experiment.

Serum chemistry analysis is slightly more amenable to high-containment laboratory studies, as many analytes are stable for prolonged periods by frozen storage, allowing analysis to be conducted at a time convenient for staff. However, the concern for generating aerosols and limited BSC space remains, and some analytes, particularly enzymes, exhibit reduced activity with freeze–thaw cycles. Point-of-care serum chemistry analyzers are available, but cost and rodent serum volumes can be prohibitive.

## Summary

While the SARS-CoV-2 pandemic has highlighted the value of pathologic assessment of preclinical models, it has also thrust many without prior high-containment laboratory experience into working in such environments and facing the challenge of collecting and assessing tissue specimens from these studies. It may not be feasible to adhere to traditional best practices due to constraints inherent to conducting work in these facilities. However, with proper planning and good communication of the goals of the study and questions to be addressed, technical modifications can be made to minimize the use of animals and maximize scientific value (Table 1). Such dialogue within the community positions us to further develop models and techniques that will be used to respond to further emerging pathogens and future pandemics.

**Table 1.** Summary of important considerations when conducting high-containment animal studies.

Prior to study start	During study	Following study termination
High-containment facility space/capabilities Availability of cage space Training/availability of high-containment personnel Availability of reagents and equipment in the high-containment facility Approval status of biosafety, inactivation, and animal use protocols Additional select agent requirements (if applicable) Minimizing animal numbersAlterations in tissue collection protocols	Enhanced PPE (respiratory protection, cut-resistant gloves, etc) Reagents/supplies needed Sharps handling/disposal Methods of anesthesia, sedation, and euthanasia Clinical monitoring frequency Experimental and humane end points Time to process each animal Sample storage/stability before transport	Fixation method(s) to conduct desired downstream analyses Decontamination and sample inactivation SOPs for removal from high-containment facility Waste management Material transfer and disposal documentation for sample transfer to other entities Additional select agent requirements (if applicable)

Abbreviation: PPE, personal protective equipment; SOP, standard operating protocol.
